# Effects of different exercise modalities on inflammatory markers in the obese and overweight populations: unraveling the mystery of exercise and inflammation

**DOI:** 10.3389/fphys.2024.1405094

**Published:** 2024-06-12

**Authors:** Yongqing Guo, Haonan Qian, Xianyang Xin, Qinlong Liu

**Affiliations:** ^1^ Capital University of Physical Education and Sports, Beijing, China; ^2^ Department of Physical Education, Hanyang University, Seoul, Republic of Korea

**Keywords:** obese and overweight, exercise modalities, inflammatory factors, meta-analysis, chronic inflammation

## Abstract

In the realm of obesity and overweight, the risk of chronic diseases significantly escalates, closely intertwined with inflammatory factors. Research suggests that specific exercise interventions, particularly aerobic exercise and resistance exercise, can have beneficial effects on inflammation levels. However, debates persist regarding the actual impact of exercise in the obese and overweight population. We employed meta-analysis research methods and searched the China National Knowledge Infrastructure Wanfang Data, PubMed, and Web of Science databases to gather controlled experiments on the effects of resistance exercise or aerobic exercise on C-reactive protein (CRP), interleukin-6 (IL-6), and tumor necrosis factor-alpha (TNF-α). Two researchers independently conducted literature screening and data extraction. The quality of the literature was assessed according to the Cochrane Handbook standards, and subgroup analyses of CRP, IL-6, and TNF-α were performed using RevMan 5.4 software. Through quantitative synthesis of results from 22 selected studies encompassing a total of 1,135 research subjects, this study systematically explored the specific regulatory effects of different exercise modalities on inflammatory markers in the obese and overweight population. The findings indicate that both aerobic exercise and resistance exercise effectively reduce CRP levels in obese individuals, with aerobic exercise demonstrating a more pronounced effect. Aerobic exercise also significantly lowers IL-6 levels, while the impact of resistance exercise on IL-6 is relatively minor. However, in terms of reducing TNF-α levels, neither modality appears to exert a significant effect. Overall, exercise, especially aerobic exercise, emerges as a positive regulator of inflammatory markers in the context of obesity and overweight.

## 1 Introduction

Obesity is widely recognized as a chronic metabolic disorder, with its prevalence showing a continuous upward trend globally (Ng et al., 2014). There is a close association between obesity and cardiovascular diseases, diabetes, fatty liver, and certain cancers. This association is not merely due to the mechanical burden of excess weight but more so because of the chronic inflammatory state caused by obesity. Insulin resistance and adipocyte proliferation caused by obesity lead to the secretion of inflammatory mediators by adipocytes, such as Interleukin-6 (IL-6), Tumor Necrosis Factor-alpha (TNF-α), and C-reactive protein (CRP). These three biomarkers play a crucial role in reflecting the body’s inflammatory state and overall health. When it comes to acute inflammatory markers, C-reactive protein (CRP) undoubtedly stands out as the most common. Levels of CRP rise rapidly during inflammation or tissue damage. Numerous studies have shown a strong correlation between elevated CRP levels and various diseases, including but not limited to cardiovascular diseases, diabetes, and obesity. Therefore, CRP is widely used to assess patients’ inflammatory status and disease risk (Ridker et al., 2002). On the one hand, Interleukin-6 (IL-6) is a crucial cytokine that plays a key role in the body’s immune response and inflammation processes. On the other hand, elevated levels of IL-6 are typically associated with chronic inflammatory conditions, such as obesity and metabolic syndrome. Additionally, IL-6 is involved in regulating blood sugar and lipid metabolism, closely linking it to the occurrence and development of various chronic diseases (Krogh-Madsen et al., 2004; Pedersen and Febbraio, 2008a). Lastly, High levels of TNF-α have been found to be associated with chronic diseases such as rheumatoid arthritis, inflammatory bowel disease, and cardiovascular diseases. Furthermore, TNF-α is also involved in the pathogenesis of obesity and metabolic syndrome, affecting the body’s metabolic status by regulating inflammation in adipose tissue and insulin resistance (Hotamisligil et al., 1993). At the same time, during adolescence and early adulthood (11–30 years old), the body undergoes significant physiological changes, including hormonal fluctuations and rapid growth (Patton et al., 2016). Generally, the overall level of inflammation during this stage is relatively low. Sex hormones such as estrogen and testosterone can influence the production and activity of inflammatory cytokines such as IL-6 and TNF-α. Additionally, young individuals typically have healthier lifestyles, including higher levels of physical activity and healthier dietary habits, which contribute to lower levels of inflammation (Park et al., 2012). During middle age (31–60 years old), the body’s physiological functions remain relatively stable, but there may be an onset of age-related chronic diseases. With advancing age, an increase in body fat content and a decline in metabolic function lead to a rise in low-level chronic inflammation. Individuals in this age group may experience higher levels of inflammatory markers due to factors such as insulin resistance and metabolic syndrome (Hotamisligil, 2006). Additionally, increased work stress and family responsibilities may also contribute to higher levels of inflammation (Kivimäki et al., 2000). In the elderly (65 years old and above), susceptibility to chronic diseases increases, and physiological function declines further, leading to a further increase in inflammation levels. As age increases, the deposition of abdominal fat and the prevalence of chronic diseases such as diabetes, cardiovascular disease, and arthritis rise, resulting in elevated systemic inflammation levels. Immune senescence is a significant factor, as changes in the aging immune system lead to the emergence of chronic low-level inflammation (Franceschi and Campisi, 2014). Current research on whether different types of exercise can reduce inflammatory marker levels remains highly debated. On one hand, a series of studies support the positive impact of exercise on reducing inflammatory markers in obese individuals. These exercise interventions include various forms such as aerobic exercise, resistance exercise, and others. These studies suggest that moderate and sustained exercise can modulate the immune system, alleviate chronic inflammation, and help improve metabolic conditions associated with obesity. For example, Chantal A. Vella et al. (2017) (Vella et al., 2017) conducted an 8-week trial intervention using aerobic exercise on obese individuals, with results indicating a significant reduction in CRP and IL-6, although TNF-α showed no significant change. Wang Chaoxun (2006) (Wang et al., 2006) conducted an 8-week intervention using aerobic exercise in obese and overweight individuals, revealing no significant changes in IL-6, but a decrease in CRP and TNF-α levels. Crisieli M. Tpmeleri (2016) (Tomeleri et al., 2016) employed anaerobic exercise in an 8-week trial intervention on obese or overweight individuals, demonstrating a significant decrease in CRP, IL-6, and TNF-α. On the other hand, a substantial body of research results indicates a lack of significant impact of exercise on inflammatory markers in obese and overweight populations. Aaron S. Keelly (2009) (Kelly et al., 2007a) and others conducted an 8-week aerobic exercise intervention on overweight individuals, with results showing no significant changes in CRP, IL-6, and TNF-α. Man Gyoon Lee (2012) (Lee et al., 2012) and colleagues conducted a 16-week aerobic exercise intervention in obese individuals, with no significant changes in CRP, IL-6, and TNF-α. Chen Qiong (2015) (Chen et al., 2015) utilized different types of resistance exercise for intervention in overweight individuals, resulting in a slight increase without significant reduction in the three markers. These experimental results are highly contentious, likely influenced by factors such as study design, sample characteristics, exercise intensity, measurement methods of inflammatory markers. Therefore, it is crucial to organize existing literature data to derive relatively consistent and rational research conclusions. This study aims to retrieve relevant previous research, employ meta-analysis methods, and systematically integrate and analyze data from the literature. The comprehensive evaluation of the impact of aerobic and resistance exercises on inflammatory marker levels in obese and overweight populations is intended to provide a reliable foundation for the development of more effective health intervention strategies for obese and overweight individuals.

## 2 Materials and methods

### 2.1 Literature sources and retrieval

The literature search covered the following databases: PubMed, Web of Science (utilizing both free-text and MeSH terms), China National Knowledge Infrastructure (CNKI), and Wanfang Database. The search spanned the last 2 decades and included the following search terms: inflammatory factors,” “C-reactive protein,” “tumor necrosis factor-alpha,” “interleukin-6,” “exercise,” “obesity,” “overweight,” “aerobic exercise,” “resistance exercise.” To ensure comprehensive retrieval, the search criteria also included “combination exercise. Language restrictions were set for Chinese and English, and the retrieval was concluded by 10 December 2023, adopting a retrospective method to ensure comprehensiveness.

### 2.2 Inclusion and exclusion criteria

Meta-analysis, a method pivotal in evidence-based medicine, leverages primary literature for statistical analysis to comprehend clinical issues. To elevate the quality of the included literature, this study adhered to the PICOS principle from Cochrane systematic reviews. PICOS encapsulates Population, Intervention, Comparison, Outcome, and Study design, long regarded as the gold standard for Meta-analysis. The inclusion and exclusion criteria based on PICOS for this study are as follows:

P (Population): Obese(BMI:>30.0) or overweight (BMI:25.0–29.9) individuals; Age:10–70age

I (Intervention): Intervention using aerobic or resistance exercise

C (Comparison): All types of comparisons/control, including self-control design

O (Outcome): CRP, IL-6, TNF-α

S (Study design): All types of comparisons/control, including self-control design

### 2.3 Article screening and data extraction

Researcher Guo and Xin extract basic information from the literature, including titles, publication years, author names, study designs, characteristics of study subjects, sample sizes, intervention details, sample sources, outcome indicators, and result information. Data extraction is conducted by Guo. When there is disagreement about including or excluding an article, researchers Guo and Xin discuss it, seeking consensus. If they cannot reach an agreement, they consult Liu and Qian. Liu makes the final decision based on their arguments.

Study design: Includes self-control and randomized controlled trials.

Characteristics of study subjects: Age, gender, BMI.

Intervention details: Experimental interventions using aerobic or resistance exercise, including intervention methods, duration, intensity, and primary exercise forms.

Outcome indicators and result information: CRP, IL-6, TNF-α.

### 2.4 Quality assessment

In Meta-analysis, the utilization of effective quality assessment methods allows for the evaluation of whether the study design and processes of included literature meet scientific standards. This study opted for the Cochrane standard for assessment, evaluating the risk of random sequence generation, allocation concealment, blinding of participants and personnel, blinding of outcome assessment, incomplete outcome data, selective reporting, and other biases for each item. A clear risk assessment was conducted for each item, categorized as high risk, low risk, or unclear, ensuring the credibility of the study and the scientific validity of the results.

### 2.5 Statistical analysis

Data analysis was conducted using Manager 5.4 (Review Manager Collaboration, 2020). Acknowledging potential differences between different experiments, the effect size in this study was represented by the Weighted Mean Difference (WMD), with a calculation of the 95% confidence interval. Heterogeneity in the study was measured using the consistency coefficient P and I2. Given statistical heterogeneity among study groups but not clinical heterogeneity, a random-effects model was applied, and subgroup analysis was performed to compare the impact of different exercise modes on inflammatory factors (CRP, IL-6, TNF-α). Sensitivity analysis was conducted to eliminate the influence of bias on the overall effect of individual studies showing differences.

## 3 Results

### 3.1 Literature search results

**FIGURE 1:**
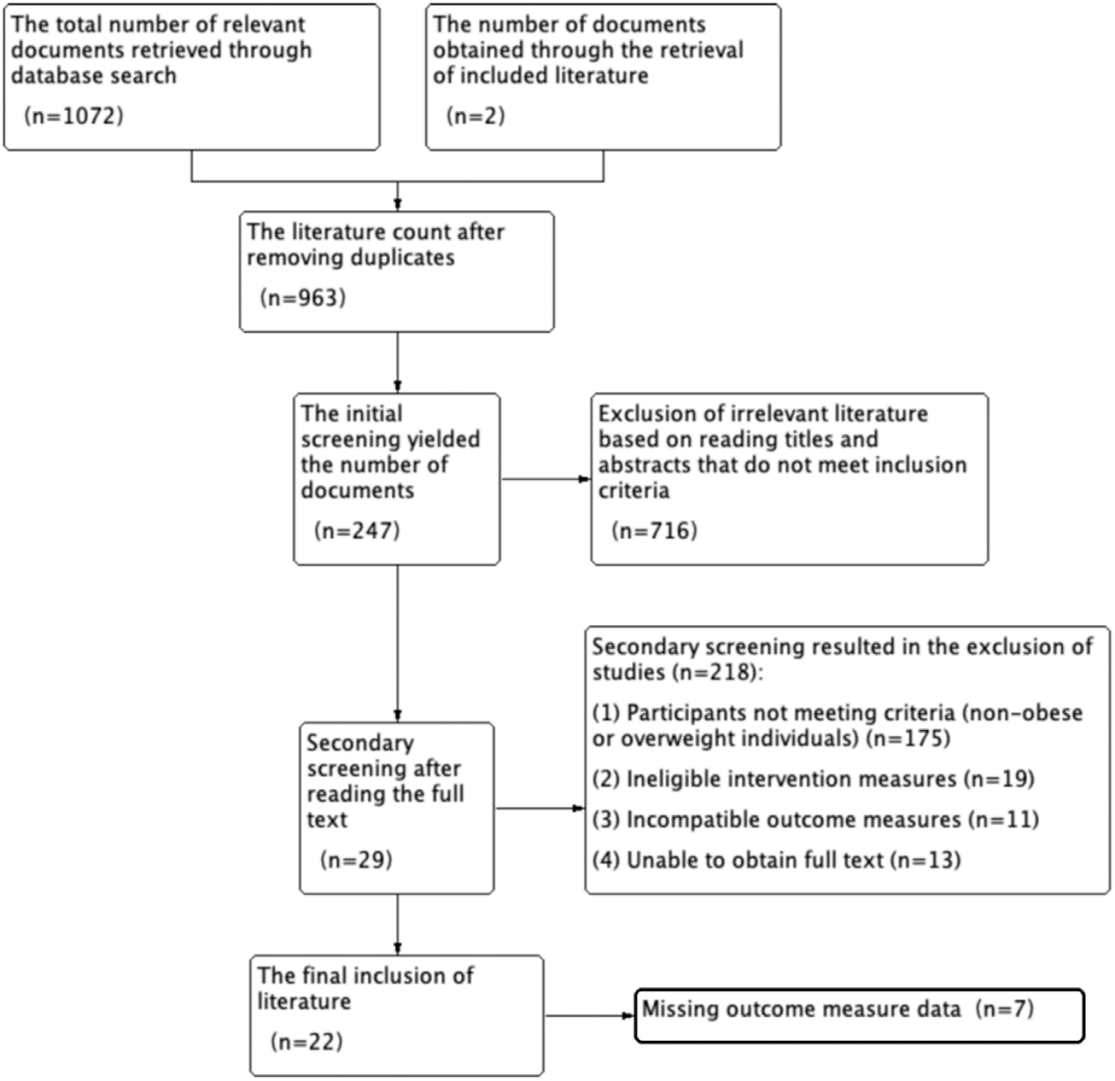
Flowchart of literature selection process.

A total of 326 articles were retrieved from the Chinese database, and 746 articles from the English database, with an additional 2 articles supplemented through references identified during the search. In total, 1,072 articles were initially retrieved. After screening, a final selection of 26 articles was included (You et al., 2004; Wang et al., 2006; Kelly et al., 2007a; Kim et al., 2007; Arsenault et al., 2009; Campbell et al., 2009; Christiansen et al., 2010; Carrillo et al., 2012; Lee et al., 2012; Ho et al., 2013; Croymans et al., 2014; Chen et al., 2015; Tomeleri et al., 2016; Vella et al., 2017; Nikseresht, 2018; Tenório et al., 2018; Wang et al., 2018; Wedell-Neergaard et al., 2019; Zhao and Liang, 2019; Chow et al., 2021; Pérez-López et al., 2022; Saeidi et al., 2023), comprising 18 in English and 4 in Chinese(All are Chinese Core Journals). The literature selection process is illustrated in Figure 1. Relevant literature was obtained through database searches (n = 1,072), with an additional 2 articles identified through references in the selected literature. After removing duplicate articles (n = 963), a thorough review of full texts and titles resulted in the inclusion of 247 studies. Subsequent screening of full texts (n = 29) led to the exclusion of 218 studies for various reasons: mismatched study subjects (n = 175), inappropriate intervention measures (n = 19), incongruent outcome indicators (n = 11), and unavailability of full texts (n = 13). A final screening of experimental data related to outcome indicators was performed (n = 7), resulting in the ultimate inclusion of 22 articles.

### 3.2 Quality assessment results

**FIGURE 2:**
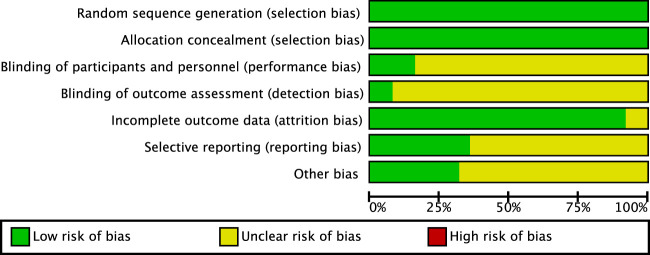
Proportional representation of risk of bias assessment in included controlled trials.

**FIGURE 3:**
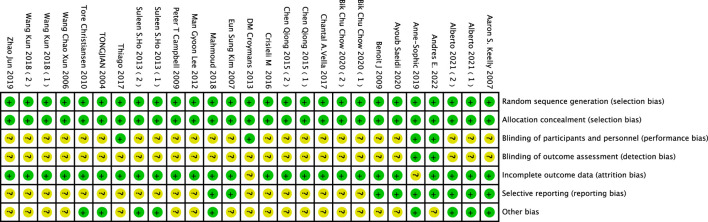
Schematic representation of risk of bias assessment for included controlled trials.

This study incorporates 22 research articles, and each article was evaluated according to Cochrane standards, as shown in Figure 2 and Figure 3. Among the 26 studies, 7 employed self-control (Christiansen et al., 2010; Carrillo et al., 2012; Chen et al., 2015; Vella et al., 2017; Tenório et al., 2018; Wang et al., 2018; Wedell-Neergaard et al., 2019) while 19 adopted random control (Wang et al., 2006; Kelly et al., 2007a; Kim et al., 2007; Arsenault et al., 2009; Campbell et al., 2009; Carrillo et al., 2012; Lee et al., 2012; Ho et al., 2013; Croymans et al., 2014; Tomeleri et al., 2016; Nikseresht, 2018; Zhao and Liang, 2019; Chow et al., 2021; Pérez-López et al., 2022; Saeidi et al., 2023). The evaluation results reveal that all included articles mention the generation and concealed allocation of random sequences, categorizing them as low risk. Additionally, 4 articles (Carrillo et al., 2012; Croymans et al., 2014; Tenório et al., 2018; Wedell-Neergaard et al., 2019) provide detailed descriptions of double-blinding between implementers and participants. Due to variations in intervention intensity or the use of placebos, detecting subtle changes in movement intensity is challenging. However, in other studies where interventions involve only two different exercises or a single exercise, implementing blinding becomes difficult, making it challenging to avoid revealing the study’s purpose. Nine studies reported participant dropouts, but all were deemed low risk as the dropouts were unrelated to the experimental intervention. Regarding outcome indicators, most articles did not explicitly specify them. Therefore, studies with unclear outcome descriptions were considered unclear.

Note:

1. Generation of random sequence (selection bias);
2. Allocation concealment (selection bias);
3. Double-blinding of implementers and participants (implementation bias);
4. Blinding in outcome assessment (detection bias);
5. Incomplete outcome data (attrition bias);
6. Selective reporting (reporting bias);
7. Other biases (excluding other important biases in the biases mentioned above).

### 3.3 Basic characteristics of included studies

This meta-analysis includes a total of 22 studies, as shown in Table 1 and Table 2, comprising 4 published in Chinese core journals and 18 in English, spanning from 2003 to 2023. The total number of participants across these studies is 1,135, with a wide age range. Participants predominantly have elevated body fat levels, indicating characteristics of obesity and overweight. The majority of intervention durations in the included studies were 12 weeks, with only one study having an intervention lasting less than 8 weeks (Kim et al., 2007), and the longest intervention period reaching 1 year (12 months) (Campbell et al., 2009).

**TABLE 1:** Overview of basic characteristics of included literature in meta-analysis.

Author (publication year)	Research type	Gender	Age		BMI		Sample size Con/Ex
			Control group/Experimental group		Control group/Experimental group		
Shen Wang 2018(1)	self-control	Male/Female	13.21 ± 1.28	13.21 ± 1.28	30.70 ± 2.11	30.70 ± 2.11	45
Shen Wang 2018(2)	self-control	Male/Female	13.21 ± 1.28	13.21 ± 1.28	30.70 ± 2.11	30.70 ± 2.11	45
Chaoxun Wang 2006	randomized control	Male/Female	24.7 ± 5.3	22.5 ± 4.8	BMI2≥5kg/m2	BMI^2^≥5 kg/m^2^	35/32
Jun Zhao 2019	randomized control	Male	21.45 ± 1.02	21.68 ± 0.98	≥28 kg·m−2	≥28 kg·m−2	24
Qiong Chen 2015	self-control	Male	14.4 ± 3.2	14.1 ± 3.1	≥25 kg·m−2	≥25 kg·m−2	15
Qiong chen 2015	self-control	Male	14.4 ± 3.2	13.9 ± 2.2	≥25 kg·m−2	≥25 kg·m−2	15
Aaron S. Keelly 2007	randomized control	Male/Female	11.0 ± 0.71	10.8 ± 0.67	30.5 ± 2.3	32.7 ± 2.6	10/9
Eun Sung Kim 2007	randomized control	Male	17 ± 0.1	17 ± 0.1	29.50 ± 0.4	29.50 ± 0.4	12/14
Chu Chow 2020(1)	randomized control	Female	19.4 ± 0.5	19.7 ± 0.9	24.8 ± 1.7	24.2 ± 1.1	11/10
Chu Chow 2020(2)	randomized control	Female	19.4 ± 0.5	19.9 ± 0.9	24.8 ± 1.7	24.8 ± 2.6	10/10
Benoit J.Arsena 2009	randomized control	Female	57.2 ± 6.1	57.3 ± 6.6	31.9 ± 3.8	32.0 ± 5.7	82/267
Suleen S.Ho 2013(1)	randomized control	Male/Female	52 (40–66)	55 (44–62)	32.4 (26.0–48.0)	25.0–45.6	16/15
Suleen S.Ho 2013(2)	randomized control	Male/Female	52 (40–66)	52 (43–59)	32.4 (26.0–48.0)	25.8–44.6	16/16
Tpmeleri 2016	randomized control	Female	66.8 ± 3.2	69.5 ± 4.7	27.1 ± 3.8	27.8 ± 4.5	19/19
Chantal A.Vella 2017	self-control	Male/Female	28.9 ± 8.1	28.9 ± 8.1	33.1 ± 6.0	33.1 ± 6.0	9
DM Croymans 2013	randomized control	Male	(20.8–22.8)	20.8–22.8	30.9 (29.7–32.7)	33.6 (31.2–34.7)	8/28
Thiago R.S 2017	self-control	No description	15 ± 14	15 ± 14	34.87 ± 4.22 kg.m−2	34.87 ± 4.22 kg m-^2^	31
Man Gyoon Lee 2012	randomized control	Female	38.3 ± 4.9	41.6 ± 4.5	(kg·m−2) > 25	(kg·m^−2^)> 25	7/8
Anne-Sophic 2019	self-control	Male/Female	39 ± 13	39 ± 13	33.0 (4.9)	33.0 (4.9)	14
Tore Christiansen 2010	self-control	Male/Female	37.2 ± 7	37.2 ± 7	33.3 ± 4	33.3 ± 4	19
TONGJIAN 2004	randomized control	Female	57 ± 1	59 ± 1	31 ± 1.4 kg/m2	31 ± 1.4 kg/m2	17/17
Peter T.C 2009	randomized control	Female	60.9 ± 6.8	60.5 ± 7.0	30.4 ± 3.8	30.2 ± 4.0	57/47
Mahmoud 2018	randomized control	Male	40.1 ± 3.1	40.1 ± 3.1	29.8 ± 2.1	29.8 ± 2.1	10/12
Alberto 2021(1)	randomized control	Female	56.9 ± 5.8	43.1 ± 2.8	34.9 ± 6.4	37.0 ± 2.8	12/10
Alberto 2021(2)	randomized control	Female	56.9 ± 5.8	43.1 ± 2.8	34.9 ± 6.4	37.0 ± 2.8	12/10
Andres E. 2022	self-control	Male/Female	26.0 ± 4.5	26.0 ± 4.5	31.9 ± 3.3	31.9 ± 3.3	13
Ayoub Saeidi 2020	randomized control	Male	27.50 ± 9.4	27.50 ± 9.4	32.9 ± 1.2 kg/m2	32.9 ± 1.2 kg/m2	11/11

**TABLE 2:** Overview of research methodology design and outcome measures in included literature for meta-analysis.

Author (publication year)	Intervention method	Intervention intensity	Intervention frequency	Intervention duration	Sample source	Outcome measures
Shen Wang 2018(1)	AE*	4 sets at 85% of VO2max heart rate, sustained for 4 min each	3 times/week	12	Serum	CRP,TNF-α
Shen Wang 2018(2)	AE	walk	30 min/time, 3 times/week	12	Serum	CRP,TNF-α
Chaoxun Wang 2006	AE	BPM(beats/min) = 170 - age	>30 min/time 3 times/week	8	Serum	CRP,TNF-α,IL-6
Jun Zhao 2019	AE	60%–70% of maximum heart rate	60 min/time, 5time/week	12	Serum	CRP
Qiong Chen 2015	AE	Heart rate at 60% of VO2max	3time/week	8	Serum	CRP,TNF-α,IL-6
Qiong chen 2015	RE*	Circuit sets, 8–12 reps per set, 2–3 sets	3time/week	8	Serum	CRP,TNF-α,IL-6
Aaron S. Keelly 2007	AE	Gradually increase intensity and duration *	4time/week	8	Serum	CRP,IL-6,TNF-α
Eun Sung Kim 2007	AE	First 3 weeks (jumps/min) 60, next 3 weeks 90	40 min/time, 5time/week	6	No description	hs-CRP,IL-6,TNF-α
Chu Chow 2020(1)	AE	Gradually increase the number of repetitions	3time/week	12	Serum	IL_6,TNF-α
Chu Chow 2020(2)	AE	Gradually increase the number of repetitions	3time/week	12	Serum	IL_6,TNF-α
Benoit J.Arsena 2009	AE	Heart rate corresponding to 50% of peak VO2 for each female	3–4time/week	24	Plasma	CRP,IL-6,TNF-α
Suleen S.Ho 2013(1)	AE	60 %HRR	30 min/time, 5time/wk	12	Plasma	IL_6,TNF-α
Suleen S.Ho 2013(2)	RE	10-repetition maximum level, 4 sets of 8–12 repetitions	30 min/time, 5/week	12	Plasma	IL_6,TNF-α
Tpmeleri 2016	RE	8 full-body exercises at 10–15 repetition maximum (RM), for a total of 3 sets	3time/week, 20 min/time	12*	Serum	CRP,IL-6,TNF-α
Chantal A.Vella 2017	AE	5%–59% HRR	2time/week, 30 min/time,4time/wk*	8	Serum	CRP,IL-6,TNF-α
DM Croymans 2013	RE	Stage 1, Stage 2, Stage 3.*	3time/week, 1 h/time	12	Serum	CRP
Thiago R.S 2017	AE	Intensity <20% of VT1, duration >50 min, up to 350 kcal	3time/week	24	Serum	IL_6,TNF-α
Man Gyoon Lee 2012	AE	50% of VO2max, exercise intensity equivalent to kilograms of body weight	1 h/week*	14	Serum	CRP,IL-6,TNF-α
Anne-Sophic 2019	AE	treadmill walking	Not accurately described	12	No description	hs-CRP,IL-6
Tore Christiansen2010	AE	Energy expenditure of 500–600 kcal	3time/week, 60–75 min/time	12	Serum	IL_6
TONGJIAN 2004	AE	50%–55% HRR for 20 min, 65%–70% HRR for 45–60 min*	HRR, 3time/week	6mth	Plasma	IL-6,TNF-α,CRP
Peter T.C 2009	AE	(60%–75% of maximum heart rate)	≥45 min/time, 5time/week	12mth	Serum	CRP,IL-6
Mahmoud 2018	RE	Performing at different intensities (40%–95% of 1-repetition maximum)	3time/week 5-11exercises	12	Serum	IL-6
Alberto 2021(1)	AE	60 min at moderate intensity (55%–75% HRR)	3time/week, 60 min/time	12	Serum	CRP
Alberto 2021(2)	AE	60 min at moderate intensity (55%–75% HRR)	3time/week, 60 min/time	12	Serum	CRP
Andres E. 2022	RE	3 sets of 8 exercises, with a 1-repetition maximum of 70%–80%	3time/week	12	Serum	IL-6,TNF-α,CRP
Ayoub Saeidi 2020	RE	10 repetitions at 50% of 1-repetition maximum intensity, 14 repetitions, 3 sets	3time/week, 60 min/time	12	Plasma	CRP

Note:

^a^ AE:aerobic exercise, RE:resistance exercise.

^b^ Gradual intensity increase*: Vo_2_max 50%–60%, 30 min; 60%–70%, 40 min; 70%–80%, 50 min.

^c^ Phases 1, 2, and 3*: In Phase 1 (Weeks 1–2), participants completed two exercises with 12–15 repetitions, approximately 12-15RM (i.e., participants were encouraged to reach volitional fatigue/failure within 15 repetitions). In Phase 2 (Weeks 3–7), participants performed three sets of exercises with 8–12 repetitions, targeting 8-12RM., In Phase 3 (Weeks 8–12), participants completed six to eight repetitions with a load corresponding to 6-8RM., as participants adapted to the exercise overload, the resistance increased to maintain the prescribed exercise intensity.

^d^ 50%–55% HRR/20 min, 65%–70% HRR, 45–60 min*: Starting from the first week with 50%–55% HRR, for 20 min, progressing to 65%–70% HRR, for 45–60 min by the third month.

^e^ 2 times/week, 30 min/session, 4 times/week*: 2 times/week for 3 weeks (supervised sessions), 30 min/session, 4 times/week for 5 weeks (unsupervised).

^f^ 1 h/week*: First 4 weeks—13.5 METs┐h/week; Middle 5 weeks - 18 METs┐h/week; Last 5 weeks—22.5 METs┐h/week.

Aerobic exercise interventions primarily involved cycling and running/walking, while resistance exercise interventions mainly utilized resistance machines and bench presses. Thirteen studies incorporated running and cycling in aerobic exercise interventions, and five studies focused on bench presses in resistance exercise interventions. Some studies also included variations in aerobic and resistance exercise modes. This meta-analysis investigated the impact of aerobic exercise or resistance exercise on CRP levels, incorporating a total of 17 studies. Additionally, the effects of aerobic exercise and resistance exercise on IL-6 and TNF-α levels were examined, including 17 studies and 14 studies, respectively.

In these studies, the intensity and dose of aerobic exercise varied, typically involving moderate to high intensity, performed 3–5 times per week, with each session lasting 30–60 min. The intensity and dose of resistance exercise also varied, generally involving moderate to high intensity, performed 2–3 times per week, with each session comprising 8–12 different resistance exercises, each set repeated 8–12 times.

(7) 12-week intervention period: 8 weeks of experimentation +4 weeks of measurement, totaling 12 weeks

### 3.4 Meta-analysis

#### 3.4.1 CRP

**FIGURE 4:**
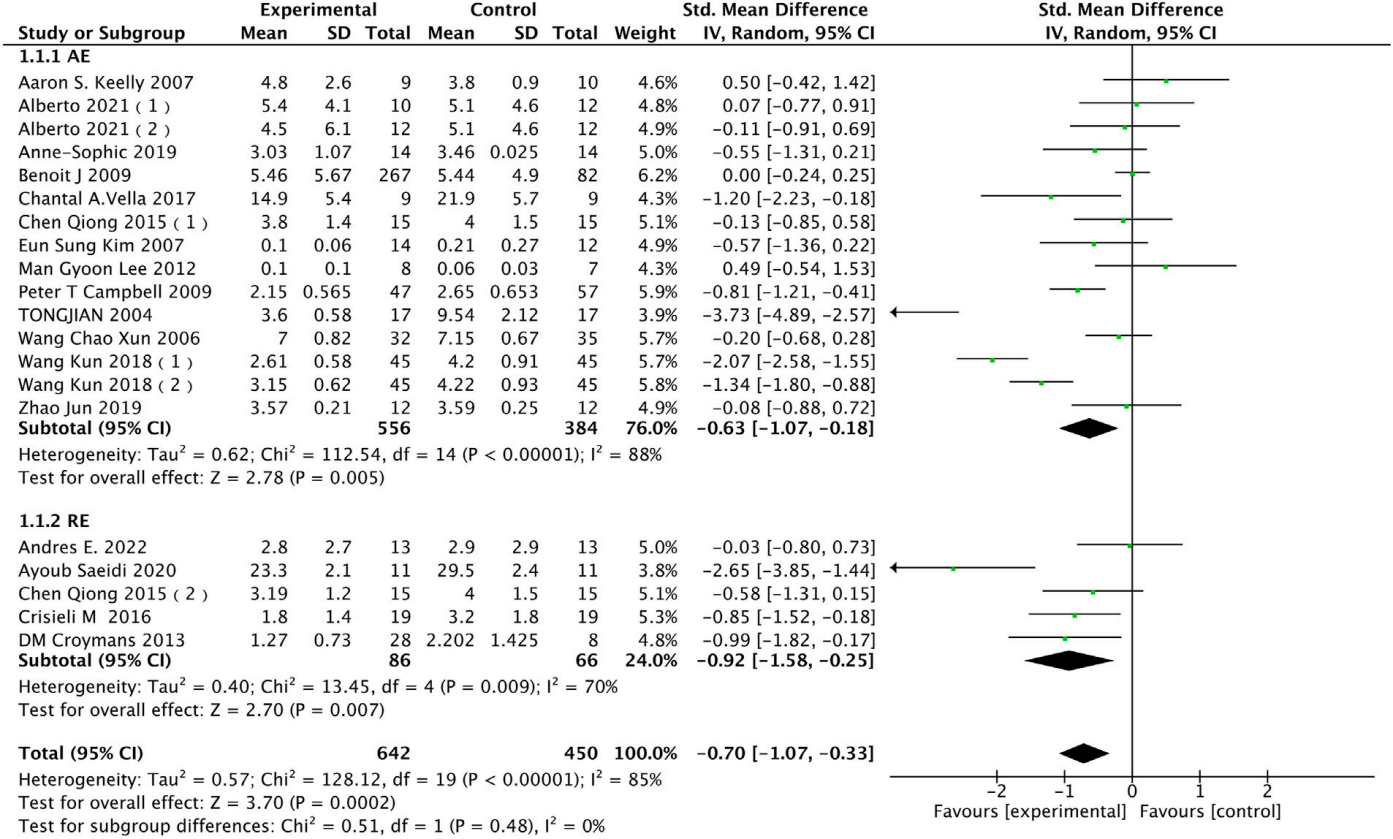
Subgroup analysis of the influence of various exercise modalities on CRP in the obese population.

CRP is a common inflammatory marker, and elevated levels of CRP areclosely associated with increased risk of atherosclerosis and cardiovascular events. In Figure 4, in terms of CRP data, 17 studies involving a total of 959 participants were included in the analysis. A random-effects model was employed to study the impact of different exercise modalities on CRP levels in the obese and overweight population, revealing high heterogeneity among the studies (I^2^ = 85%, *p* < 0.00001). Using a random-effects model, the pooled effect size was [SMD = −0.70, 95% CI = −1.07 to −0.33 (*p* = 0.0002)], indicating that exercise can reduce CRP levels in the obese and overweight population to a certain extent. Subgroup analysis showed that AE [SMD = −0.63, 95% CI = −1.07 to −0.18, (*p* = 0.005)] and RE [SMD = −0.92, 95% CI = −1.58 to −0.25, (*p* = 0.007)] significantly lowered CRP levels in the obese and overweight population. The subgroup analysis results indicated that both aerobic and resistance exercises could significantly reduce CRP levels in the obese and overweight population, with the former showing a more pronounced regulatory effect on CRP.

#### 3.4.2 IL-6

**FIGURE 5:**
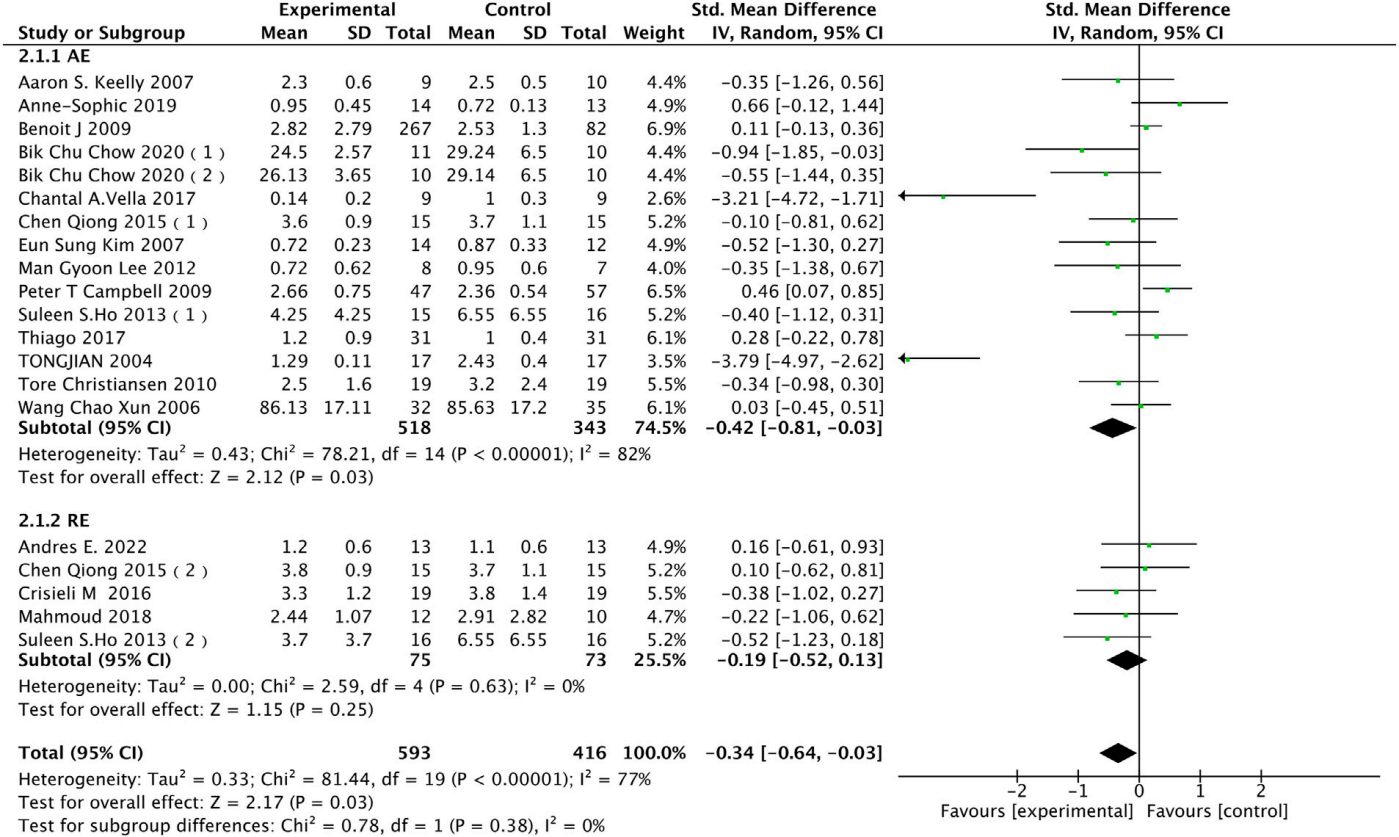
Subgroup analysis of the effects of different exercise modalities on IL-6 in the obese population.

In the context of IL-6, as shown in Figure 5, this study includes 17 research articles with a total of 917 participants. Analyzing the impact of different exercise modalities on IL-6 levels in the obese and overweight population reveals high heterogeneity among the studies (I^2^ = 77%, *p* < 0.00001). Using a random-effects model, the pooled effect size is [SMD = −0.34, 95% CI = −0.64 to −0.03 (*p* = 0.03)], indicating that exercise can moderately reduce IL-6 levels in the obese and overweight population. The subgroup analysis results indicate that AE can effectively reduce IL-6 levels in the obese and overweight population(SMD = −0.42, 95% CI = −0.81 to −0.03, *p* = 0.03), while the impact of RE on IL-6 levels in the obese population is not significant(SMD = −0.19, 95% CI = −0.52 to 0.13, (*p* = 0.25)).

Caption: Compared to pre-exercise intervention levels, AE effectively reduces interleukin-6 levels, while the impact of RE on interleukin-6 levels reduction is not significant (*p* = 0.03/*p* = 0.25).

#### 3.4.3 TNF-α

**FIGURE 6:**
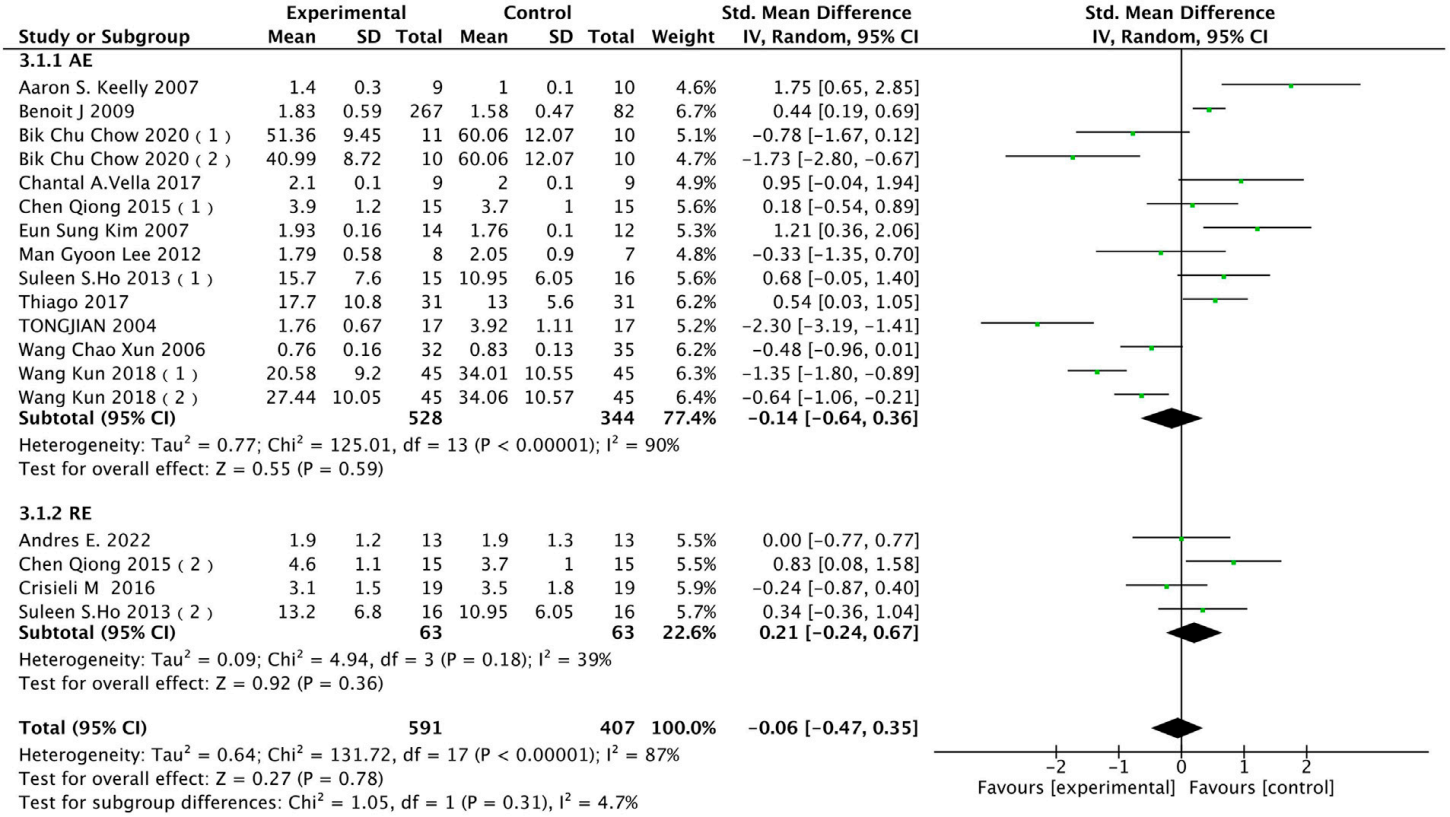
Subgroup analysis of the impact of Diverse exercise modalities on TNF-α in the obese population.

Tumor necrosis factor-alpha (TNF-α) is an inflammatory mediator typically associated with inflammation and immune responses. Exercise has complex effects on the immune system and inflammatory responses, often considered to have a regulatory impact on certain inflammation markers. As shown in Figure 6, in terms of TNF-α data, 14 studies involving a total of 835 participants were included in the analysis. Using a random-effects model to study the impact of different exercise modalities on TNF-α levels in the obese and overweight population, high heterogeneity was observed among the studies (I^2^ = 87%, *p* < 0.00001). Applying the random-effects model, the pooled effect size was [SMD = −0.06, 95% CI = −0.47 to 0.35 (*p* = 0.78)], indicating that exercise has no significant effect on reducing TNF-α levels in the obese and overweight population. Subgroup analysis showed that AE [SMD = −0.14, 95% CI = −0.64 to 0.36, (*p* = 0.59)] and RE [SMD = 0.21, 95% CI = −0.24 to 0.67, (*p* = 0.36)]. There was no significant effect on reducing TNF-α levels in the obese and overweight population.

Caption: Compared to pre-exercise intervention levels, both AE and RE show no significant effect on reducing TNF-α levels (*p* = 0.59/*p* = 0.36).

### 3.5 Publication bias and sensitivity analysis

**FIGURE 7:**
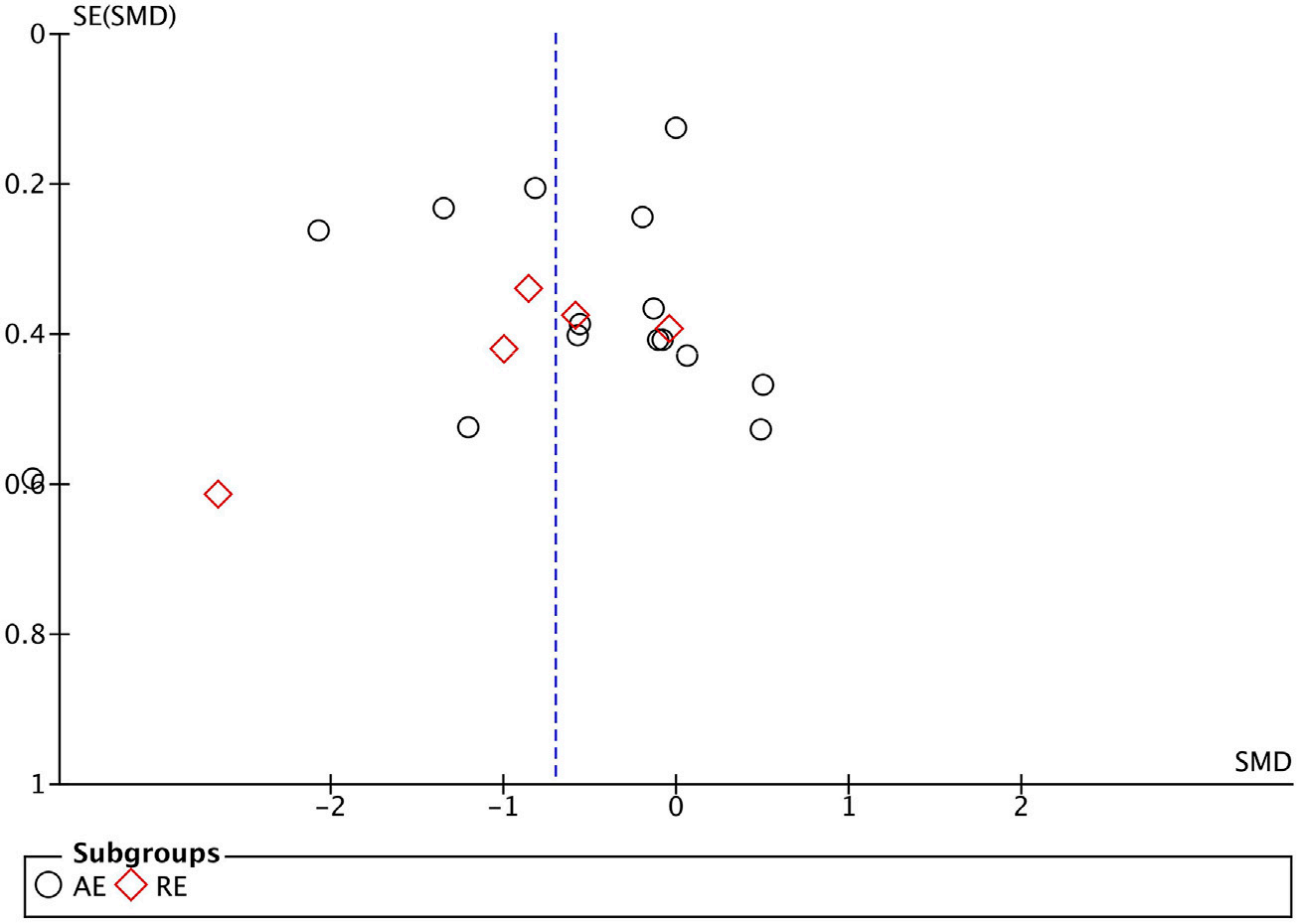
Funnel Plot Illustrating the Impact of Different Exercise Modalities on CRP in the obese and overweight Population.

**FIGURE 8:**
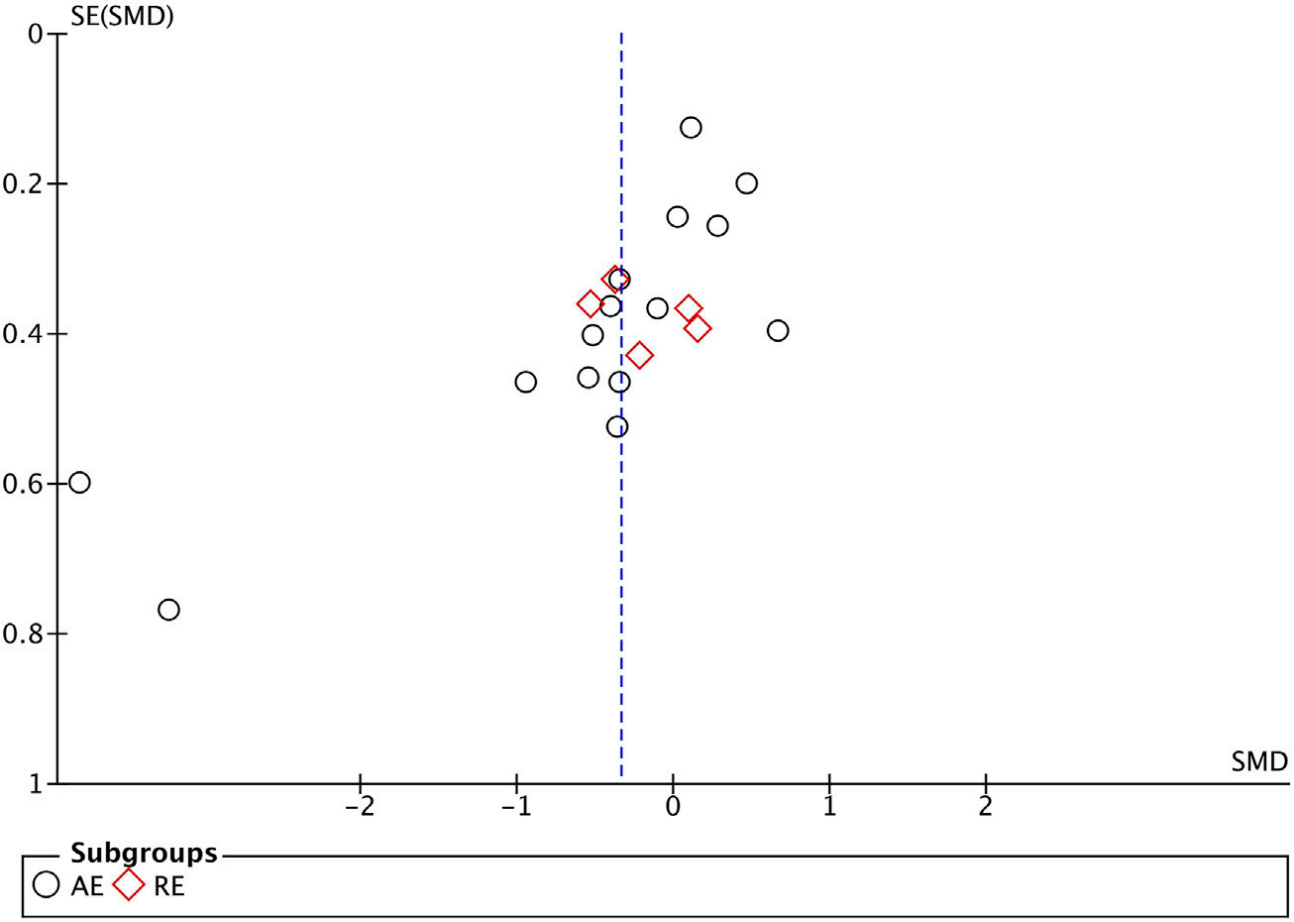
Funnel Plot illustrating the impact of different exercise modalities on IL-6 in the obese and overweight population.

**FIGURE 9:**
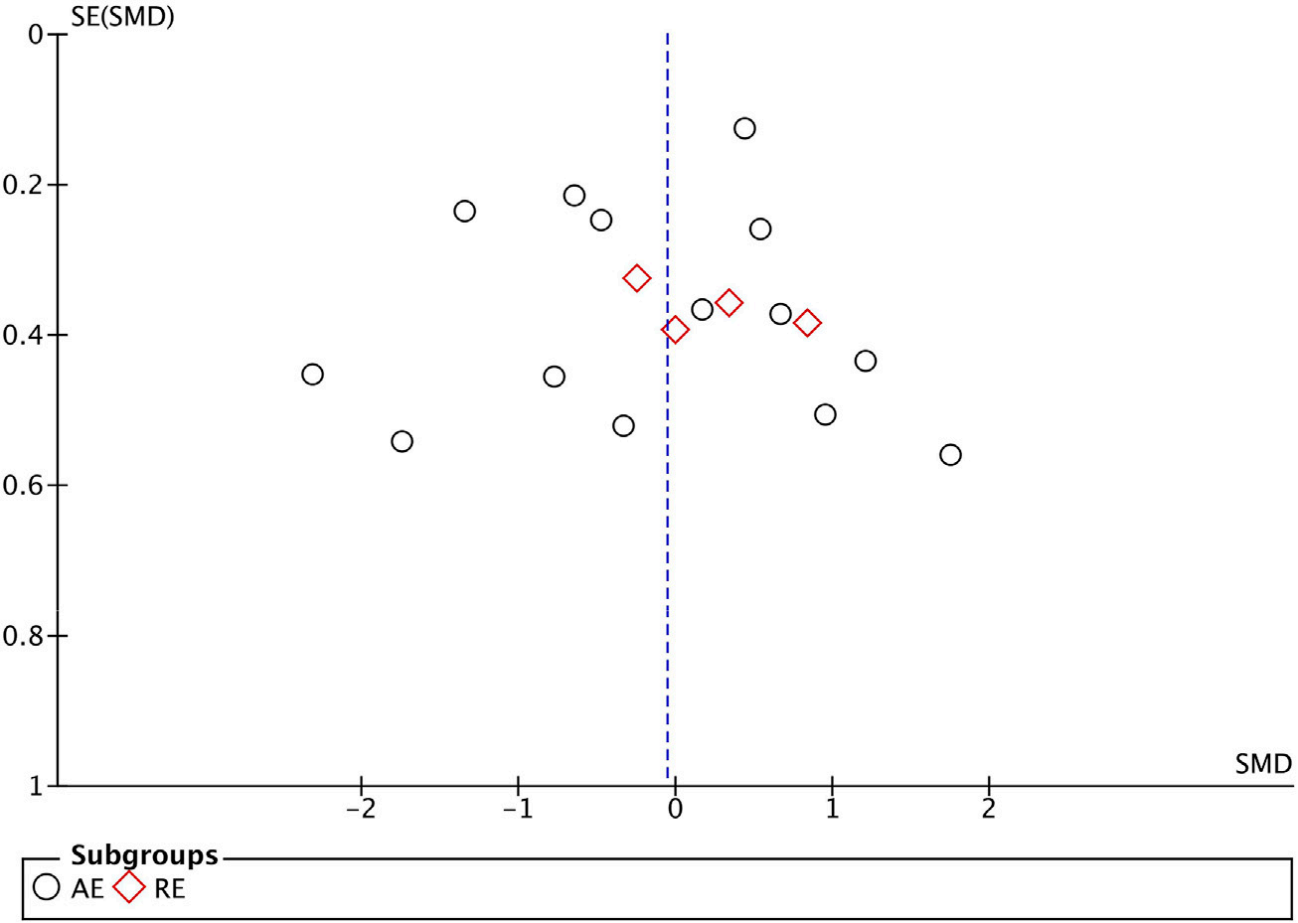
Funnel Plot Illustrating the Impact of Various Exercise Modes on TNF-α in the obese and overweight Population.

The evaluation of publication bias in the Revman software is presented through a funnel plot. In this study, funnel plots were constructed with the standard error (logarithm of SMD) on the *x*-axis and the standard error of SMD (SE(SMD)) on the *y*-axis. Funnel plots of the effects of aerobic exercise on CRP, IL-6, and TNF-α are presented below. The publication bias of different exercise modes on CRP in obese and overweight populations is shown in Figure 7: the majority of the scattered points are concentrated in the middle of the graph, distributed relatively symmetrically. However, two studies are located in the bottom left corner, suggesting potential publication bias. Sensitivity analysis was conducted, and after removing some literature (You et al., 2004; Wang et al., 2018), the results showed that the for aerobic exercise (AE) decreased to over 50%, while resistance exercise (RE) decreased to over 20%, with no significant difference in results observed. The funnel plot for IL-6 (Figure 8) demonstrates that the scattered points of each study are concentrated, but two study is located in the bottom left corner, indicating potential publication bias. After literature exclusion (You et al., 2004; Vella et al., 2017), the for AE decreased to over 50%, with no change in results observed. The TNF-α funnel plot (Figure 9) shows that the studies are concentrated with significant symmetry on both sides, indicating no apparent publication bias. Regarding the bias of the above indicators, we isolated and excluded these studies before re-conducting the meta-analysis. Compared to previous research results, no significant changes were found. The effect sizes still had statistical significance, demonstrating the reliability of the previous analysis conclusions. If you are preparing this for submission to an English journal, I can help you further refine and polish it.

### 3.6 Summary of meta-analysis results

Initially, looking at the overall results, both aerobic exercise and resistance exercise appear to reduce CRP levels in obese and overweight populations, with aerobic exercise demonstrating a more pronounced effect in lowering CRP. Additionally, aerobic exercise effectively lowers IL-6 levels in obese and overweight individuals, while resistance exercise shows no significant impact on reducing IL-6. Both aerobic and resistance exercises seem to have no significant effect on lowering TNF-α levels in obese and overweight populations. Despite the possibility of high heterogeneity among the included studies, sensitivity analysis indicates that the results remain stable and reliable. In the summary of the inflammatory factor studies, no single study was found to exert a disproportionately large influence on the overall results. The inclusion criteria for the studies were met uniformly, and heterogeneity may stem from variations in exercise intensity and volume or potential age and gender differences. Therefore, this study employed the standardized mean difference (SMD) and a random-effects model to minimize the impact of literature heterogeneity on the results.

## 4 Discussion

It is widely recognized that individuals with obesity and overweight, especially those with excessive accumulation of visceral fat, often generate pro-inflammatory cytokines, which may significantly trigger systemic inflammation (Fried et al., 1998; Harkins et al., 2004). Mild chronic inflammation is often closely associated with excessive caloric intake and a lack of physical activity. Tumor necrosis factor-alpha (TNF-α), interleukin-6 (IL-6), and C-reactive protein (CRP) are closely linked to this condition. Meta-analysis of inflammatory marker data from study subjects suggests that exercise significantly reduces CRP and IL-6 levels in obese populations, aligning with previous research. Surprisingly, exercise shows no significant impact on lowering TNF-α levels, which may involve differences in biological mechanisms or study design. Subgroup analysis of different exercise modalities indicates that aerobic exercise can significantly reduce CRP and IL-6 levels in obese and overweight individuals. Some studies have indicated that aerobic exercise can increase the activity of the vagus nerve, thereby enhancing anti-inflammatory effects (Tracey, 2002), a study conducted by Kevin Tracey’s laboratory precisely illustrates this point. Aerobic exercise can increase the activity of the vagus nerve, thus reducing inflammation. This mechanism may be achieved through the neuro-immune regulation between the vagus nerve and the inflammatory response. As reported by Taaffe et al., IL-6 and CRP decrease with increased moderate and high-intensity physical activity (Taaffe et al., 2000). Interestingly, the meta-analysis results reveal that aerobic exercise has a more pronounced effect on reducing CRP levels compared to resistance exercise, which may be attributed to several key physiological mechanisms. Firstly, aerobic exercise can improve blood glucose control and increase insulin sensitivity, significantly reducing insulin resistance and consequently lowering inflammation levels, such as Colberg (2016) and Pedersen (2015) (Pedersen and Febbraio, 2012; Colberg, 2016). Additionally, aerobic exercise influences the inflammatory state by regulating hormone levels, such as reducing tumor necrosis factor-alpha (TNF-α) and interleukin-1β (IL-1β), while promoting the production of more anti-inflammatory hormones, such as interleukin-10 (IL-10). This helps to balance the activity of the immune system, thereby reducing the inflammatory response (Pedersen and Febbraio, 2008b). Other studies, such as Del Rosso 2023 Obes Rev, indicate that resistance exercise (RE) also impacts IL-6 levels. Good vascular health is also one of the significant effects of aerobic exercise. Through various physiological mechanisms such as increasing cardiac contractility, improving cardiac output, and increasing the release of nitric oxide by endothelial cells, aerobic exercise can improve endothelial function, increase vascular elasticity, reduce the risk of atherosclerosis and inflammation, decrease vascular inflammatory responses and endothelial cell damage, thus lowering CRP levels (Green et al., 1985). Furthermore, aerobic exercise promotes the oxidative metabolism of fatty acids within fat cells, reducing the release of interleukin-6 (IL-6) from fat cells, further lowering CRP levels. The combined effects of these physiological responses may explain why aerobic exercise demonstrates more significant reductions in CRP and IL-6 levels. Additionally, the analysis results show that resistance exercise has minimal fluctuations in IL-6 levels, but overall, the actual impact is not significant, consistent with the findings of Wang Chaoxun (2006) (Wang et al., 2006). Furthermore, aerobic exercise is typically associated with a high number of submaximal muscle contractions, whereas resistance exercise emphasizes short-duration, high-intensity muscle contractions. This difference might result in insufficient mechanical stimulation during resistance exercise to significantly impact the release of interleukin-6 (IL-6). Additionally, aerobic exercise is commonly accompanied by substantial oxidative glycogen utilization, while resistance exercise may rely more on the phosphagen system. IL-6 production is closely linked to glycogen consumption and metabolic status, potentially leading to a relatively minor influence of resistance exercise on IL-6. It is noteworthy that neither aerobic nor resistance exercise exhibits a significant regulatory effect on TNF-α levels in obese and overweight populations. Specifically, the study by Colbert et al. (Harris, 2004) found a negative correlation between individual activity levels and CRP, iIL-6, and TNF-α. Higher activity levels were associated with lower levels of inflammatory markers, but the study also discovered that the correlation between activity levels and TNF-α became non-significant after intervening with obesity factors. In a subsequent study by Beavers et al. (2010) (Beavers et al., 2010), examining a broader range of inflammatory biomarkers in the same cohort, TNF-α remained unaffected. Similarly, in the study by Chen Qiong (2015) (Chen et al., 2015), TNF-α levels slightly increased in obese and overweight individuals after 8 weeks of resistance exercise, possibly due to induced muscle microdamage and fatigue (Cheung et al., 2003). Moreover, CRP and IL-6 exhibit higher sensitivity compared to TNF-α. The lack of TNF-α elevation may also be associated with the duration of exercise, as most studies (Hammett et al., 2004; Kohut et al., 2006; Nicklas et al., 2008; Vieira et al., 2009) show a reduction in systemic inflammatory markers after at least 24 weeks of intervention, while shorter intervention durations (less than 24 weeks) have no significant impact on inflammatory markers ^[^ (Colbert et al., 2004; Martins et al., 2010)^]^.

Furthermore, within the confines of this study, there is notable heterogeneity observed in the levels of CRP, TNF-α, and IL-6. This complex scenario may be attributed to an array of factors, including a substantial age range, gender diversities, and variances in intervention intensity and duration. These multifaceted factors could introduce latent variables that exert influence on the observed outcomes. Primarily, the diversity in age emerges as a pivotal factor contributing significantly to the discerned heightened heterogeneity. Participants spanning different age cohorts may find themselves in disparate physiological states. For instance, elderly individuals undergo the rigors of physiological aging, resulting in a discernible decline in immune system functionality (Larbi et al., 2008). In contrast, their younger counterparts may revel in a state of physiological vitality, maintaining a relatively robust immune profile. Such variances in physiological states are likely to impinge upon baseline levels of inflammatory markers and the nuanced response to exercise interventions (Woods et al., 2012). Furthermore, the nuanced age-related variations in the sensitivity of the anti-inflammatory system may contribute to disparate responses to identical interventions. As individuals progress in age, there is a gradual attenuation of immune function, instigating the progression of inflammatory processes (Colbert et al., 2004). Even under ostensibly healthy conditions, older individuals manifest a 2-4-fold elevation in pro-inflammatory cytokines and acute-phase proteins when compared to their younger counterparts (Bruunsgaard and Pedersen, 2003). Simultaneously, gender-based disparities emerge as another prominent source of heightened heterogeneity. Within the In CHIANTI study, the engagement in walking activities for 2–4 h per week demonstrated an association with diminished CRP levels among males and a concomitant reduction in IL-6 levels among females (Elosua et al., 2005).

Albert et al., in their thorough investigation, found that even after thorough adjustments for BMI and other cardiovascular risk factors, a residual negative correlation between CRP and male physical activity persisted, while the relationship between physical activity and female CRP levels remained subtle (Albert et al., 2004). Furthermore, insights garnered from a comprehensive study conducted by Aaron S. Kelly (2007)indicate that a condensed 8-week regimen of physical activity might potentially suffer from brevity or subdued intensity, rendering it challenging to incite discernible enhancements in adipose factors and oxidative stress reactions (Kelly et al., 2007a), This kind of brief or low-intensity exercise may lead to reduced exercise effectiveness. A shorter exercise period may fail to fully activate the metabolic pathways within fat cells, thus inadequately reducing the release of fat factors and limiting metabolic changes in the body, thereby restricting its impact on inflammation, and may even fail to induce sufficient physiological changes (Kim et al., 2006). Generally, physical exercise requires sufficient time to trigger adaptive responses in the body, especially in improving metabolic function and reducing inflammation. Additionally, a shorter exercise duration may also fail to provide enough mechanical stimulation, which is particularly important in resistance exercise, as moderate muscle stimulation can promote muscle adaptive responses (Kelly et al., 2007b). Conversely, a longer exercise period may provide more opportunities to gradually increase exercise loads and allow the body to adapt to more physiological changes. In long-term exercise, the body may gradually adjust metabolic pathways, enhance anti-inflammatory mechanisms, and improve tissue health (Gleeson et al., 2011). Therefore, compared to short-term exercise, long-term exercise may produce more sustainable and significant effects (Schoenfeld et al., 2017). Compelling evidence substantiates the dichotomous impact of exercise on inflammation, whereby heightened intensity in acute exercise may precipitate muscular damage, particularly in instances of prolonged and intense workouts, thereby eliciting inflammatory cell infiltration and elevated levels of muscle-specific creatine kinase subtypes (Clarkson and Hubal, 2002). Noteworthy, Taaffe et al. brought to light an intriguing inverse correlation between walking speed and IL-6 and CRP levels (Shoelson and Lee, 2006). Consequently, disparities in exercise intensity and duration could feasibly contribute to the augmented heterogeneity observed in research outcomes.

## 5 Conclusion

Based on evidence from experimental interventions employing both pre-post self-control and randomized control trials, it is indicated that both aerobic exercise and resistance exercise can reduce CRP levels in obese individuals, with aerobic exercise showing a more pronounced effect. Additionally, aerobic exercise has been demonstrated to effectively lower IL-6 levels, whereas resistance exercise does not exhibit a significant impact on IL-6 levels in obese populations. In the literature reviewed, neither aerobic nor resistance exercise shows a significant reduction in IL-6 and TNF-α levels in the human body. Further support for these findings could be derived from additional high-quality studies in the future.

## 6 Limitations of this study

This study followed the prescribed procedures outlined by the PRISMA guidelines. However, due to the wide standard deviation in the age range of participants included in the literature, it was not possible to conduct subgroup analyses regarding the impact of different age groups on inflammatory factors. The sensitivity of the anti-inflammatory system, influenced by age, resulted in varying outcomes for the same indicators following exercise interventions, introducing a certain level of heterogeneity in the results. Moreover, differences in intervention intensity and duration may have exposed the study to the influence of undisclosed factors, potentially contributing to an elevated level of heterogeneity.
